# The Church and the River

**Published:** 1889-06-29

**Authors:** 


### The Church and the River.

It was an interesting sight we witnessed from the Thames embankment a month or two ago, when the Bishop of London dedicated to the service of God the new steam launch that had been acquired for the work of the Thames Church Mission. The weather, to quote a hackneyed phrase, was very inclement—that is to say, the rain was coming down in torrents—and as the proceedings were to take place in the open air the prospect was not encouraging. But Dr. Temple is not the man to be easily turned from the path of duty. He donned his robes in a pier shelter, and proceeded on board the launch, closely followed by his footman, who held over his lordship a large carriage umbrella. Surrounding the Bishop, at the stern of the vessel, were a number of clergy in surplices, while the congregation was made up of friends and supporters of the Thames Church Mission; the ladies, amongst whom was Mrs. Temple, rivalling the gentlemen in their courage to face the storm on the open deck. A small crowd looked on from the Embankment and Waterloo Bridge in wondering astonishment at the strange spectacle.

It may well have been that the unpropitious weather was in some degree symbolical of the difficulties the agents of the Thames Church Mission have sometimes to encounter in their daily visitation of the ships. The attitude of the sailors, however, towards the Mission is very different now to what it once was. It is not so very long since that the missionaries were rebuffed by the men, often with rudeness, and sometimes with violence. This very rarely happens now. The difficulties are of another character. In the course of their work the missionaries come across all sorts and conditions of men; some openly profess infidelity, others are indifferent and prefer a life of sin; others, again are gospel-hardened—that is to say, they have heard the "good news" so often and rejected it that it falls now as seed upon stony ground. These three classes of men present difficult problems, but it is encouraging to know that they are in many cases being satisfactorily solved.

The district, or parish, as it is sometimes called, of the Mission extends from Richmond to the Nore, a distance of more than 70 miles, and it is computed that there are afloat within this area somewhere about 300,000 souls. The peculiar circumstances of these men is worth more than a passing thought. In far too many instances they are by the very nature of their calling cut off from all the means of grace. They have no church and no ministry, and unless they can be reached when they are in port there is little reason to suppose that they will ever come under good influences. No one can well deny that in the past these men were shamefully neglected; they felt, as one "old salt" put it to the writer a short time ago, that no one cared for them. But this reproach has happily been rolled away, and there is today probably more work being done for the sailor by religious agencies than for many other class of men similarly situated.

It will be useful to enquire what is being done in the Port of London. The Thames Church Mission employs three chaplains and twenty lay agents, a number far too small to do anything like all the work that is ready to their hands. But this is not their fault, nor does the blame rest with the authorities of the Mission. The need for an extension of the work is abundantly recognised, and it is for those who feel that the poor sailor should have some share in the spiritual privileges that they enjoy to supply the requisite means. It must be remembered that the Mission has not one halfpenny of endowment; it is entirely dependent on voluntary contributions. Perhaps some day an opportunity will open for the Church in her corporate capacity to do something to supply the needs of the seafaring population; until then, individual members of the Church whose hearts are stirred must do the work. But small though the staff be, the Thames Church Mission can show a good record. The statistics for 1888 are not, we understand, available for public use; we hear, however, that they will show an increase in almost every department of labour over the previous year. Here are some of the figures for 1887: 79,059 visits to individuals, 16,326 visits to barges, 19,839 visits to ships and steamers, and 2,385 visits to foreign vessels; 538,260 English and 18,452 foreign tracts distributed, also 1,277 English and 2,813 foreign "portions" of Scripture and 17,573 copies of the New Testament; 641 Bibles and 5,845 New Testaments in English and 105 Bibles and 104 New Testaments in foreign languages sold; 450 Prayer-books either sold or given away; 603 total abstinence pledges taken; library bags placed on 269 additional ships, and last, but by no means least, 5,065 services held with an aggregate attendance of 130,727 persons.

The full spiritual result of all this external labour will never be known on this side of eternity, but the Mission is not without strong evidence, even now, that its labours are, by the blessing of God, having an appreciable effect upon the lives of the men amongst whom it labours. "If the ladies and gentlemen who send you on the river," recently remarked an inspector of the Thames Police to the missionary who works amongst the bargemen, "only knew the difference in our sailors, fishermen, and bargemen since I joined the force 15 years ago they would hardly credit it. They could never believe what a different class of men we have now, and do you know what I believe has led to this change for the better? The Thames Church Mission!" Equally striking testimony on the same point has been borne by the Bishop of Bedford, than whom there is hardly a keener judge of men and things. "I have been long enough in the East-end," he said lately, "to see some proof of the good work of this society and other kindred institutions. . . . Through their instrumentality seamen have often become more sober, more thrifty, and now, I believe, at the same time more godly." It would be impossible to go afloat in any district of the river with any one of the agents of the Thames Church Mission without coming across many a bright and pious Christian on board ship. The conduct of some of the men at the service the missionary generally tries to hold in the foc's'le during dinner hour is very encouraging. They are reverent, earnest, and devout, and testify in no uncertain terms to the influence for good the work of the Mission has had upon them.

But, as we pointed out above, although there may be, and undoubtedly is, much to encourage, there is still more to depress. Where we find one Christian, there are sometimes as many as half-a-dozen who are living in careless indifference of, if not in open hostility to, religious things. It is in order that the Mission may continue its beneficent labours amongst these, as well as seek to enlarge the border of its operations, that the committee appeal to their fellow-Churchmen for that sympathy and support which Churchmen are never really backward in extending to a good cause. H. C. H.

